# New Scalarane Sesterterpenoids from the Formosan Sponge *Ircinia felix*

**DOI:** 10.3390/md13074296

**Published:** 2015-07-14

**Authors:** Ya-Yuan Lai, Mei-Chin Lu, Li-Hsueh Wang, Jih-Jung Chen, Lee-Shing Fang, Yang-Chang Wu, Ping-Jyun Sung

**Affiliations:** 1 Graduate Institute of Marine Biology, National Dong Hwa University, Pingtung 944, Taiwan; E-Mails: spire0123456@yahoo.com.tw (Y.-Y.L.); jinx6609@nmmba.gov.tw (M.-C.L.); wanglh@nmmba.gov.tw (L.-H.W.); 2 National Museum of Marine Biology and Aquarium, Pingtung 944, Taiwan; 3 Department of Pharmacy & Graduate Institute of Pharmaceutical Technology, Tajen University, Pingtung 907, Taiwan; E-Mail: jjchen@tajen.edu.tw; 4 Department of Sport, Health and Leisure, Cheng Shiu University, Kaohsiung 833, Taiwan; E-Mail: lsfang@csu.edu.tw; 5 School of Pharmacy, College of Pharmacy, China Medical University, Taichung 404, Taiwan; 6 Chinese Medicine Research and Development Center, China Medical University Hospital, Taichung 404, Taiwan; 7 Center for Molecular Medicine, China Medical University Hospital, Taichung 404, Taiwan; 8 Graduate Institute of Natural Products, Kaohsiung Medical University, Kaohsiung 807, Taiwan; 9 Department of Marine Biotechnology and Resources, Asia-Pacific Ocean Research Center, National Sun Yat-sen University, Kaohsiung 804, Taiwan

**Keywords:** *Ircinia felix*, sponge, scalarane, sesterterpenoid, cytotoxicity

## Abstract

Five new scalarane sesterterpenoids, felixins A–E (**1**–**5**), were isolated from the Formosan sponge *Ircinia felix*. The structures of scalaranes **1**–**5** were elucidated on the basis of spectroscopic analysis. Cytotoxicity of scalaranes **1**–**5** against the proliferation of a limited panel of tumor cell lines was evaluated.

## 1. Introduction

**Figure 1:**
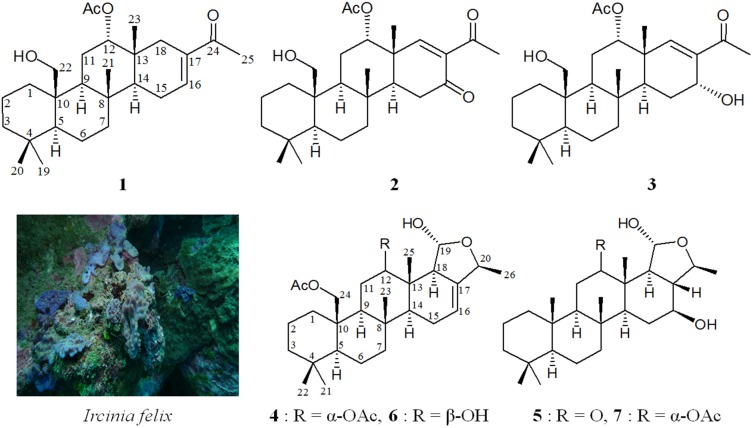
The sponge *Ircinia felix* and the structures of felixins A–E (**1**–**5**) and 12-deacetyl-23-acetoxy-20-methyl-12-*epi*-deoxyscalarin (**6**) and scalarane **7**.

Marine sponges belonging to the genus *Ircinia* (family Irciniidae, order Dictyoceratida, class Demospongiae, phylum Porifera) have been proven to be not only an important source of various interesting natural substances [1,2,3,4,5], but have also played an interesting role in marine ecology [6,7,8,9,10] and medicinal use [11,12]. In continuing research aimed at the discovery of new bioactive substances from marine organisms, an organic extract of the sponge identified as *Ircinia felix* (Duchassaing and Michelotti, 1864) (Figure 1) exhibited cytotoxicity toward MOLT-4 (human acute lymphoblastic leukemia) tumor cells (IC_50_ < 6.25 μg/mL). We isolated five new scalarane sesterterpenoids, felixins A–E (**1**–**5**) from this organism (Figure 1). In this paper, we deal with the isolation, structure determination, and cytotoxicity of scalaranes **1**–**5**.

## 2. Results and Discussion

Felixin A (**1**) was isolated as a white powder and the molecular formula for this compound was determined to be C_27_H_42_O_4_ (seven unsaturations) using HRESIMS at *m*/*z* 453.29773 [M + Na]^+^ (calcd for C_27_H_42_O_4_ + Na, 453.29753). Comparison of the ^13^C NMR and DEPT data with the molecular formula indicated there must be an exchangeable proton, which required the presence of a hydroxy group. The IR spectrum of **1** showed strong bands at 3480, 1731 and 1662 cm^−1^, consistent with the presence of hydroxy, ester and α,β-unsaturated ketone groups. The ^13^C NMR and DEPT spectral data showed that this compound has 27 carbons (Table 1), including six methyls, nine sp^3^ methylenes (including an oxymethylene), four sp^3^ methines (including an oxymethine), four sp^3^ quaternary carbons, an sp^2^ methine and three sp^2^ quaternary carbons (including two carbonyls). Based on the ^1^H and ^13^C NMR spectra (Table 1), **1** was found to possess an acetoxy group (δ_H_ 2.08, 3H × s; δ_C_ 170.2, C; 21.5, CH_3_) and a ketonic carbonyl (δ_C_ 199.1, C-24). An additional unsaturated functionality was indicated by ^13^C resonances at δ_C_ 139.4 (CH-16) and 137.7 (C-17), suggesting the presence of a trisubstituted olefin. Thus, from the above data, three degrees of unsaturation were accounted for and **1** was identified as a tetracyclic sesterterpenoid analogue.

**Table 1:** ^1^H (400 MHz, CDCl_3_) and ^13^C (100 MHz, CDCl_3_) NMR data and ^1^H–^1^H COSY and HMBC correlations for scalarane **1**.

Position	δ_H_ (*J* in Hz)	δ_C_, Multiple	^1^H–^1^H COSY	HMBC
1	2.09 m; 0.52 ddd (12.8, 12.8, 3.2)	34.4, CH_2_	H_2_-2	C-3, -10, -22
2	1.51 m; 1.39 m	17.8, CH_2_	H_2_-1, H_2_-3	n. o. ^a^
3	1.43 ddd (12.8, 4.0, 4.0)	41.7, CH_2_	H_2_-2	C-1, -4, -5, -19, -20
	1.17 ddd (12.8, 12.8, 4.8)			
4		33.0, C		
5	0.97 dd (12.0, 2.0)	56.9, CH	H_2_-6	C-3, -4, -6, -7, -9, -10, -20, -22
6	1.58 m; 1.45 m	18.3, CH_2_	H-5, H_2_-7	C-5, -8
7	1.81 ddd (12, 8, 3.2, 3.2); 1.05 m	41.9, CH_2_	H_2_-6	C-21
8		37.4, C		
9	1.35 br d (13.2)	53.1, CH	H_2_-11	C-5, -7, -8, -10, -11, -12, -14, -21, -22
10		41.8, C		
11	2.17 m; 1.96 m	25.2, CH_2_	H-9, H-12	C-9, -13
12	4.72 dd (3.6, 2.0)	77.1, CH	H_2_-11	C-9, -14
13		35.8, C		
14	1.56 m	48.0, CH	H_2_-15	C-7, -8, -13, -15, -21, -23
15	2.34 m; 2.22 m	24.0, CH_2_	H-14, H-16	C-16, -17
16	6.86 m	139.4, CH	H_2_-15	C-14, -24
17		137.7, C		
18	2.22 m; 1.92 m	35.1, CH_2_		C-13, -14, -16, -17, -23, -24
19	0.87 s	33.8, CH_3_		C-3, -4, -5, -20
20	0.77 s	21.9, CH_3_		C-3, -4, -5, -19
21	1.10 s	15.4, CH_3_		C-7, -8, -9, -14
22	4.03 d (11.6); 3.89 d (11.6)	63.0, CH_2_		C-1, -9, -10
23	0.87 s	19.6, CH_3_		C-12, -13, -14
24		199.1, C		
25	2.28 s	25.2, CH_3_		C-17, -24
12-OAc		170.2, C		
	2.08 s	21.5, CH_3_		Acetate carbonyl

^a^ n. o. = not observed.

From the ^1^H–^1^H COSY spectrum of **1** (Table 1), it was possible to establish the separate system that map out the proton sequences from H_2_-1/H_2_-2/H_2_-3, H-5/H_2_-6/H_2_-7, H-9/H_2_-11/H-12 and H-14/H_2_-15/H-16. These data, together with the key HMBC correlations between protons and quaternary carbons (Table 1), such as H_2_-3, H-5, H_3_-19, H_3_-20/C-4; H_2_-6, H-9, H-14, H_3_-21/C-8; H_2_-1, H-5, H-9, H_2_-22/C-10; H_2_-11, H-14, H_2_-18, H_3_-23/C-13; H_2_-15, H_2_-18, H_3_-25/C-17; and H-16, H_2_-18, H_3_-25/C-24, established the carbon skeleton of **1** as a 24-homo-25-norscalarane derivative [13]. The oxymethylene unit at δ_C_ 63.0 was correlated to the methylene protons at δ_H_ 4.03 and 3.89 in the HMQC spectrum. The methylene signals were ^2^*J*-correlated with C-10 (δ_C_ 41.8) and ^3^*J*-correlated with both C-1 (δ_C_ 34.4) and C-9 (δ_C_ 53.1), proving the attachment to a hydroxymethyl group at C-10 (Table 1). Thus, the remaining acetoxy group was positioned at C-12, an oxymethine (δ_H_ 4.72, δ_C_ 77.1) as indicated by analysis of the ^1^H–^1^H COSY correlations and characteristic NMR signals, although no HMBC correlation was observed between H-12 and the acetate carbonyl.

**Figure 2:**
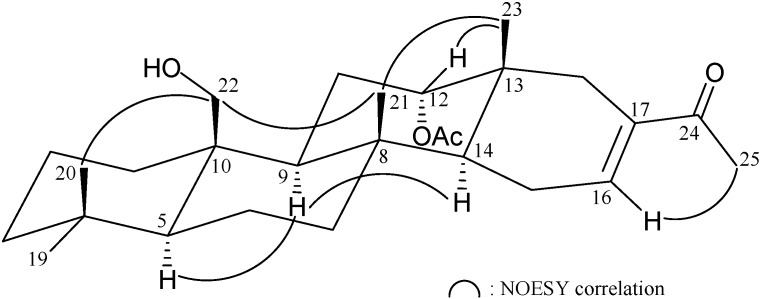
Selective NOESY correlations of **1**.

The relative stereochemistry of **1** was elucidated from the NOE interactions observed in an NOESY experiment (Figure 2). As per convention, when analyzing the stereochemistry of scalarane sesterterpenoids, H-5 and hydroxymethyl at C-10 were assigned to the α and β face, anchoring the stereochemical analysis because no correlation was found between H-5 and H_2_-22. In the NOESY experiment of **1**, H-9 showed correlations with H-5 and H-14 but not with H_3_-21 and H_2_-22. Thus, both H-9 and H-14 must also be on α face whilst Me-21 and the hydroxymethyl at C-10 must be located on the β face. Moreover, the correlations of H_3_-23/H_3_-21 and H_3_-23/H-12, indicated the β-orientation of Me-23 and H-12 attaching at C-13 and C-12, respectively. The NOESY spectrum showed a correlation of H-16 with H_3_-25, revealing the *E* geometry of the C-16/17 double bond. Based on the above findings, the structure, including the relative configuration of **1** was established unambiguously. 

**Figure 3:**
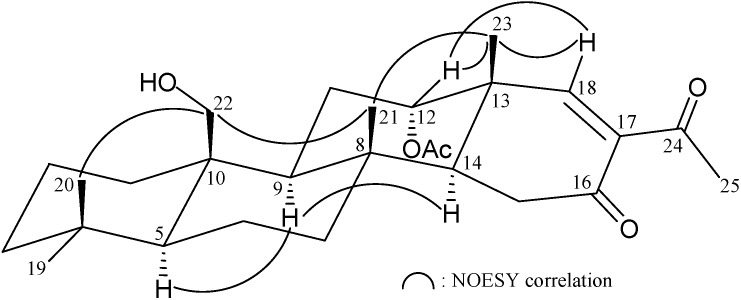
Selective NOESY correlations of **2**.

The HRESIMS of **2** (felixin B) exhibited a pseudomolecular ion peak at *m*/*z* 467.27707 [M + Na]^+^, with the molecular formula C_27_H_40_O_5_ (calcd C_27_H_40_O_5_ + Na, 467.27680), implying eight degrees of unsaturation. The IR absorptions of **2** showed the presence of hydroxy (3501 cm^−1^), ester carbonyl (1733 cm^−1^) and α,β-unsaturated ketone (1679 cm^−1^) functionalities. The ^13^C NMR and DEPT spectrum of **2** exhibited for all 27 carbons: two ketones (δ_C_ 197.9, C-24; 197.7, C-16), an ester carbonyl (δ_C_ 170.2, acetate carbonyl), a trisubstituted olefin (δ_C_ 163.9, CH-18; 136.6, C-17), an oxymethylene (δ_C_ 62.7, CH_2_-22), an oxymethine (δ_C_ 76.3, CH-12), six methyls, seven methylenes, three methines and four quaternary carbons. Both the ^13^C and ^1^H NMR data for the rings A–C portions were essentially same as those of **1**. It also contained an acetoxy (δ_H_ 2.05), an acetyl (methyl ketone, δ_H_ 2.42) and a hydroxymethyl (δ_H_ 4.04 and 3.87) groups as in **1**. Analysis of ^1^H–^1^H COSY and HMBC data (Table 2) revealed the planar structure. The same stereochemistry was shown by coupling constant and NOE data (Figure 3). The NOESY spectrum showed correlations of H-18 with H-12 and H_3_-23, revealing the *Z* geometry of the C-17/18 double bond.

**Table 2:** ^1^H (400 MHz, CDCl_3_) and ^13^C (100 MHz, CDCl_3_) NMR data and ^1^H–^1^H COSY and HMBC correlations for scalarane **2**.

Position	δ_H_ (*J* in Hz)	δ_C_, Multiple	^1^H–^1^H COSY	HMBC
1	2.11, m; 0.53 ddd (13.2, 13.2, 4.0)	34.2, CH_2_	H_2_-2	C-2, -3, -10, -22
2	1.58–1.42 m	18.3, CH_2_	H_2_-1, H_2_-3	n. o. ^a^
3	1.42 m; 1.19 m	41.6, CH_2_	H_2_-2	C-4, -20
4		33.0, C		
5	0.96 m	57.0, CH	H_2_-6	C-3, -4, -6, -7, -9, -10, -20, -22
6	1.54 m; 1.42 m	17.7, CH_2_	H-5, H_2_-7	n. o.
7	1.78 ddd (12.8, 3.2, 3.2); 1.05 m	41.0, CH_2_	H_2_-6	C-21
8		37.2, C		
9	1.31 br d (13.2)	53.1, CH	H_2_-11	C-5, -7, -8, -10, -11, -21, -22
10		41.7, C		
11	2.29 ddd (13.6, 13.6, 2.4); 2.05 m	24.9, CH_2_	H-9, H-12	n. o.
12	4.97 dd (2.8, 2.8)	76.3, CH	H_2_-11	n. o.
13		41.4, C		
14	2.11 m	48.9, CH	H_2_-15	C-8, -13, -21, -23
15	2.57–2.40 m	35.0, CH_2_	H-14	C-13, -14, -16
16		197.7, C		
17		136.6, C		
18	7.30 s	163.9, CH		C-12, -14, -17, -24
19	0.87 s	33.8, CH_3_		C-3, -4, -5, -20
20	0.76 s	21.8, CH_3_		C-3, -4, -5, -19
21	1.12 s	15.7, CH_3_		C-7, -8, -9, -14
22	4.04 d (12.0); 3.87 d (12.0)	62.7, CH_2_		C-1, -9, -10
23	1.17 s	18.4, CH_3_		C-12, -13, -14, -18
24		197.9, C		
25	2.42 s	30.6, CH_3_		C-17, -24
12-OAc		170.2, C		
	2.05 s	21.2, CH_3_		Acetate carbonyl

^a^ n. o. = not observed.

**Figure 4:**
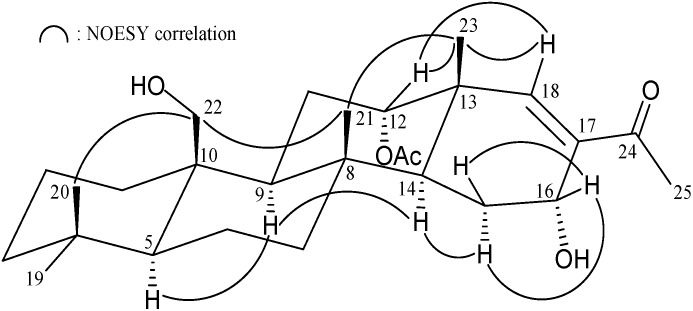
Selective NOESY correlations of **3**.

Felixin C (**3**) was isolated as a white solid. Its HRESIMS (*m*/*z* 469.29290 [M + Na]^+^) and NMR data (Table 3) established a molecular formula of C_27_H_42_O_5_ (calcd C_27_H_42_O_5_ + Na, 469.29245). The IR spectrum of **3** revealed the presence of hydroxy (*ν*_max_ 3480 cm^−^^1^) ester (*ν*_max_ 1731 cm^−^^1^) and α,β-unsaturated ketone (*ν*_max_ 1662 cm^−^^1^) groups. By comparison of NMR data of **3** with those of **2** (Table 2 and Table 3), it was found that the ketone at C-16 in **2** (δ_C_ 197.7) was replaced by a hydroxy group (δ_C_ 63.3, δ_H_ 4.55, 1H, *J* = 3.6 Hz) in **3**. Analyses of ^1^H–^1^H COSY and HMBC correlations established the planar structure of **3** (Table 3) as shown in Figure 1, which showed the C-16 positioning of the hydroxy group. Careful analysis of the NOESY spectrum of **3**, in comparison with that of **2**, allowed determination of the relative stereochemistry of A–C rings of felixin C (**3**) as shown in Figure 4. Moreover, the splitting pattern and *J*-value of proton at C-16 in **3**, combined with the interactions observed between H-16 and both of the C-15 methylene protons revealed the α-orientation of the 16-OH. Furthermore, the correlations between the olefinic proton H-18/H_3_-23 and H-18/H-12 assigned the *E*-configuration of the double bond between C-17 and C-18.

**Table 3:** ^1^H (400 MHz, CDCl_3_) and ^13^C (100 MHz, CDCl_3_) NMR data and ^1^H–^1^H COSY and HMBC correlations for scalarane **3**.

Position	δ_H_ (*J* in Hz)	δ_C_, Multiple	^1^H–^1^H COSY	HMBC
1	2.08 m; 0.57 ddd (12.8, 12.8, 3.2)	34.1, CH_2_	H_2_-2	n. o. ^a^
2	1.54 m; 1.39 m	17.8, CH_2_	H_2_-1, H_2_-3	n. o.
3	1.42 m; 1.16 m	41.7, CH_2_	H_2_-2	C-20
4		33.0, C		
5	1.02 dd (12.8, 2.4)	56.8, CH	H_2_-6	C-4, -20, -22
6	1.54 m; 1.47 m	18.4, CH_2_	H-5, H_2_-7	n. o.
7	1.88 m; 1.11 m	41.3, CH_2_	H_2_-6	C-8, -21
8		36.8, C		
9	1.45 m	53.5, CH	H_2_-11	C-10, -11, -21, -22
10		41.8, C		
11	1.96–1.81 m	25.3, CH_2_	H-9, H-12	C-8, -10, -13
12	4.97 dd (2.8, 2.8)	76.5, CH	H_2_-11	n. o.
13		41.4, C		
14	1.88 m	44.0, CH	H_2_-15	C-8, -13, -15, -16, -21, -23
15	1.88 m; 1.64 dd (14.0, 4.8)	25.3, CH_2_	H-14, H-16	C-8, -13, -16, -17
16	4.55 d (3.6)	63.3, CH	H_2_-15	C-14, -17, -18
17		138.2, C		
18	6.59 s	152.2, CH		C-12, -13, -14, -16, -24
19	0.85 s	33.8, CH_3_		C-3, -4, -5, -20
20	0.76 s	21.8, CH_3_		C-3, -4, -5, -19
21	1.06 s	16.4, CH_3_		C-7, -8, -9, -14
22	4.04 d (12.0); 3.90 d (12.0)	62.8, CH_2_		C-1, -9, -10
23	1.06 s	19.5, CH_3_		C-12, -13, -14, -18
24		201.4, C		
25	2.24 s	25.4, CH_3_		C-17, -24
12-OAc		170.9, C		
	2.04 s	21.4, CH_3_		Acetate carbonyl

^a^ n. o. = not observed.

Moreover, two deoxoscalarin-like metabolites [13], felixins D (**4**) and E (**5**) were isolated from *I. felix* in this study. Felixin D (**4**) was isolated as white powder and its molecular formula was established as C_30_H_46_O_6_ from the HRESIMS at *m*/*z* 525.31849 (calcd C_30_H_46_O_6_ + Na, 525.31866). Eight degrees of unsaturation implied by the molecular formula were ascribed to five rings, a trisubstituted double bond (δ_C_ 141.2, C-17; 114.4, CH-16) and two ester carbonyl groups (δ_C_ 171.0, 170.9, 2 × C). The ^1^H NMR spectrum showed seven methyls (δ_H_ 2.10, 2.05, 2 × 3H, s, acetate methyls; 1.26, 3H, d, *J* = 6.0 Hz, H_3_-26; 0.98, 3H, s, H_3_-23; 0.89, 3H, s, H_3_-21; 0.83, 3H, s, H_3_-22; 0.78, 3H, s, H_3_-25); an acetoxymethylene (δ_H_ 4.59, 1H, d, *J* = 12.0 Hz; 4.16, 1H, d, *J* = 12.0 Hz, H_2_-24); three oxymethines (δ_H_ 5.21, 1H, d, *J* = 3.2 Hz, H-19; 4.91, 1H, dd, *J* = 3.2, 2.4 Hz, H-12; 4.62, 1H, br s, H-20); and an olefinic proton (δ_H_ 5.35, 1H, br s, H-16). The ^13^C NMR and DEPT spectra exhibited 30 signals, including seven methyls, eight sp^3^ methylenes (including an oxymethylene), seven sp^3^ methines (including three oxymethines), an sp^2^ methine, four sp^3^ quaternary carbons and three sp^2^ quaternary carbons (including two ester carbonyls). A typical sesterterpenoid carbons system bearing an acetoxymethylene and four methyl groups along rings A–D could be established by the HMBC correlations from the acetoxymethylene (CH_2_-24) and four methyl groups (Me-21, 22, 23 and 25) to the associated carbons and a deoxoscalarin skeleton could be obtained on the basis of further HMBC and ^1^H–^1^H COSY correlations (Table 4). The ^1^H–^1^H COSY correlations between H-18/H-19 and H-20/H_3_-26 and the HMBC correlations from H-19/C-20 and H_3_-26/C-17, -20, allowed the establishment of the hemiacetal ring E.

**Figure 5:**
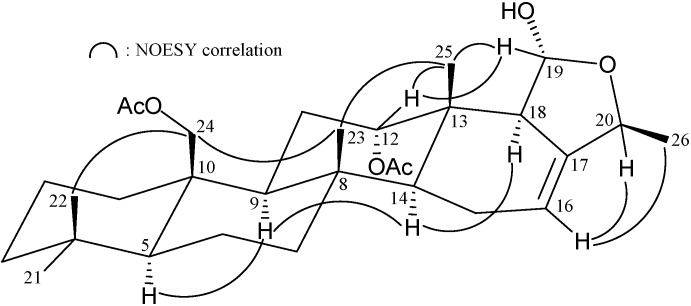
Selective NOESY correlations of **4**.

The relative stereochemistry of **4** was elucidated from the interactions observed in an NOESY experiment (Figure 5). In the NOESY experiment of **1**, H-9 showed correlations with H-5 and H-14, but not with H_3_-23 and H_2_-24 at C-10. Thus, both H-5 and H-14 must be on α face whilst Me-23 and the acetoxymethylene at C-10 must be located on the β face. The correlations of H_3_-25 with H_3_-23 and H-12 indicated the β-orientation of Me-25 and H-12. H-18 correlated with H-14, but not with H-19, and H-19 correlated with H-12 and H_3_-25, assuming that H-18 and H-19 were α- and β-oriented, respectively. H-16 showed correlations with H-20 and H_3_-26, but not with H-18, revealing the *E* geometry of the C-16/17 double bond. It was found that the structure of **4** was similar with that of a known scalarane, 12-deacetyl-23-acetoxy-20-methyl-12-*epi*-deoxo- scalarin (**6**) [14], excepting the β-hydroxy group at C-12 in **6** was replaced by an α-acetoxy group in **4**. The relative configuration of C-20 chiral carbon in **4** was elucidated by comparison the NMR data of CH-20 (δ_H_ 4.62, 1H, m; δ_C_ 74.0) of **4** with those of **6** (δ_H_ 4.67, 1H, m; δ_C_ 74.5), indicating H-20 in **4** was α-oriented. 

**Table 4:** ^1^H (400 MHz, CDCl_3_) and ^13^C (100 MHz, CDCl_3_) NMR data and ^1^H–^1^H COSY and HMBC correlations for scalarane **4**.

Position	δ_H_ (*J* in Hz)	δ_C_, Multiple	^1^H–^1^H COSY	HMBC
1	1.98 dd (13.2, 2.4)	34.7, CH_2_	H_2_-2	C-5, -9, -24
	0.57 ddd (13.2, 13.2, 2.4)			
2	1.56 m; 1.43 m	18.2, CH_2_	H_2_-1, H_2_-3	C-4
3	1.46 m; 1.15 m	41.6, CH_2_	H_2_-2	C-2, -4, -21, -22
4		33.0, C		
5	1.04 dd (12.8, 2.0)	56.8, CH	H_2_-6	C-3, -4, -6, -7, -9, -10, -21, -22, -24
6	1.56 m; 1.38 dd (13.6, 3.2)	17.9, CH_2_	H-5, H_2_-7	n. o. ^a^
7	1.79 ddd (12.8, 3.2, 3.2); 1.12 m	41.7, CH_2_	H_2_-6	C-5, -8, -9, -14, -23
8		37.7, C		
9	1.46 m	52.9, CH	H_2_-11	C-1, -5, -8, -10, -11, -12, -14, -23, -24
10		40.1, C		
11	2.05–1.89 m	25.1, CH_2_	H-9, H-12	C-8
12	4.91 dd (3.2, 2.4)	74.6, CH	H_2_-11	C-9, -14, acetate carbonyl
13		36.9, C		
14	1.64 dd (11.2, 6.4)	50.4, CH	H_2_-15	C-7, -8, -9, -13, -15, -18, -25
15	2.16 m; 1.97 m	22.9, CH_2_	H-14, H-16	C-8
16	5.35 br s	114.4, CH	H_2_-15	n. o.
17		141.2, C		
18	2.82 br s	54.7, CH	H-19	n. o.
19	5.21 d (3.2)	96.7, CH	H-18	C-20
20	4.62 m	74.0, CH	H_3_-26	n. o.
21	0.89 s	33.7, CH_3_		C-3, -4, -5, -22
22	0.83 s	21.9, CH_3_		C-3, -5, -21
23	0.98 s	15.4, CH_3_		C-7, -9, -14
24	4.59 d (12.0); 4.16 d (12.0)	64.9, CH_2_		C-1, -9, -10, acetate carbonyl
25	0.78 s	14.7, CH_3_		C-12, -14, -18
26	1.26 d (6.0)	17.6, CH_3_	H-20	C-17, -20
12-OAc		170.9, C		
	2.10 s	21.5, CH_3_		Acetate carbonyl
23-OAc		171.0, C		
	2.05 s	21.2, CH_3_		Acetate carbonyl

^a^ n. o. = not observed.

**Figure 6:**
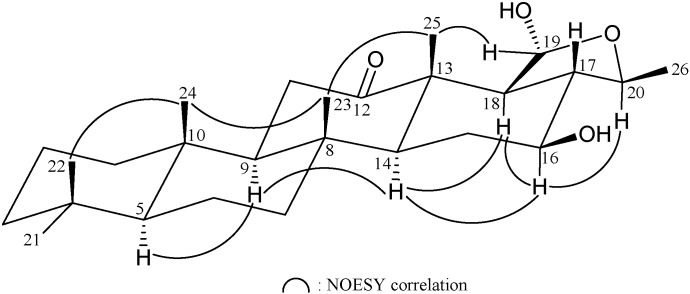
Selective NOESY correlations of **5**.

The HRESIMS of **5** (felixin E) exhibited a pseudomolecular ion peak at *m*/*z* 441.29739 [M + Na]^+^, with the molecular formula C_26_H_42_O_4_ (calcd C_26_H_42_O_4_ + Na, 441.29753), implying six degrees of unsaturation. The IR absorptions of **5** showed the presence of hydroxy (3421 cm^−1^) and ketone (1701 cm^−1^) functionalities. The ^13^C NMR and DEPT spectrum of **5** exhibited for all 26 carbons: a ketone (δ_C_ 219.0, C-12), a hemiacetal (δ_C_ 97.1, CH-19), two oxymethines (δ_C_ 78.1, CH-20; 72.0, CH-16), six methyls, seven methylenes, five methines, and four quaternary carbons (Table 5). The NMR data of **5** were similar with those of **4**, except for the acetoxymethylene group at C-10 and acetoxy group at C-12 in **4** were replaced by a methyl and a ketone group in **5**, respectively. The C-16/17 trisubstituted olefin in **4** was replaced by a hydroxy group at C-16 in **5**. The stereochemical configuration was identical to that of other scalarane sesterterpenes based on NOESY cross-peaks at H-5/H-9, H-9/H-14, H-14/H-16, H-14/H-18, H-16/H-18, H-16/H-20, H-19/H_3_-25, H_3_-22/H_3_-24, H_3_-23/H_3_-24 and H_3_-23/H_3_-25 (Figure 6). Furthermore, it was found that the structure of **5** was similar with that of known scalarane **7** [15], excepting the 12α-acetoxy group in **7** was replaced by a ketone group in **5**. 

**Table 5:** ^1^H (400 MHz, CDCl_3_) and ^13^C (100 MHz, CDCl_3_) NMR data and ^1^H–^1^H COSY and HMBC correlations for scalarane **5**.

Position	δ_H_ (*J* in Hz)	δ_C_, Multiple	^1^H–^1^H COSY	HMBC
1	1.56 m; 0.76 m	39.3 CH_2_	H_2_-2	C-5
2	1.64–1.34 m	18.3, CH_2_	H_2_-1, H_2_-3	n. o. ^a^
3	1.80 m; 1.38 m	41.6, CH_2_	H_2_-2	n. o.
4		33.3, C		
5	0.94 m	56.5, CH	H_2_-6	C-4
6	1.64–1.34 m	18.1, CH_2_	H-5, H_2_-7	n. o.
7	1.81 m; 0.94 dd (13.2, 4.0)	41.7, CH_2_	H_2_-6	C-5
8		37.8, C		
9	1.19 m	61.4, CH	H_2_-11	C-8, -12, -14, -23
10		38.2, C		
11	2.70 dd (14.0, 13.2); 2.32 dd (13.2, 2.4)	35.3, CH_2_	H-9	C-8, -9, -12
12		219.0, C		
13		51.2, C		
14	1.21 m	59.2, CH	H_2_-15	C-12, -18
15	1.95 ddd (12.8, 4.4, 2.4); 1.41 m	30.8, CH_2_	H-14, H-16	C-13
16	3.55 ddd (10.4, 10.4, 4.8)	72.0, CH	H_2_-15, H-17	n. o.
17	1.62 m	53.0, CH	H-16, H-18, H-20	n. o.
18	1.86 m	59.2, CH	H-17, H-19	C-13, -16, -19, -25
19	5.31 d (6.0)	97.1, CH	H-18	C-18, -20
20	4.10 qd (6.0, 3.2)	78.1, CH	H-17, H_3_-26	n. o.
21	0.85 s	33.2, CH_3_		C-3, -4, -5, -22
22	0.82 s	21.3, CH_3_		C-4, -21
23	1.06 s	16.9, CH_3_		C-7, -8, -9, -14
24	0.87 s	15.6, CH_3_		C-10
25	1.24 s	15.3, CH_3_		C-12, -13, -14, -18
26	1.38 d (6.0)	20.5, CH_3_	H-20	C-17, -20

^a^ n. o. = not observed.

The cytotoxicity of compounds **1**–**5** against MOLT-4 (human acute lymphoblastic leukemia), SUP-T1 (human T-cell lymphoblastic lymphoma), DLD-1 (human colorectal adenocarcinoma), LNCaP (human prostatic carcinoma), T-47D (human ductal carcinoma) and MCF7 (human breast adenocarcinoma) tumor cells are shown in Table 6. The results showed that compounds **1**–**5** were found to exhibit cytotoxicity against DLD-1 tumor cells. By comparison with the structures and cytotoxicity of scalaranes **2** and **3**, implying that the presence of 16-ketone would enhance the activity.

**Table 6:** Cytotoxic data of scalarane sesterterpenoids **1**–**5**.

Compounds	Cell Lines IC_50_ (μM)					
	MOLT-4	SUP-T1	DLD-1	LNCaP	T-47D	MCF7
**1**	NA ^b^	NA	10.9	24.3	NA	NA
**2**	14.9	27.1	8.5	NA	32.2	23.0
**3**	18.5	NA	15.0	NA	NA	NA
**4**	12.8	31.6	7.9	21.5	20.2	NA
**5**	14.0	31.1	7.2	NA	22.7	24.3
Doxorubicin ^a^	0.02	0.09	0.64	0.02	0.09	0.79

^a^ Doxorubicin was used as a positive control; ^b^ NA = not active at 20 μg/mL for 72 h.

## 3. Experimental Section

### 3.1. General Experimental Procedures

Optical rotation values were measured with a Jasco P-1010 digital polarimeter (Japan Spectroscopic Corporation, Tokyo, Japan). IR spectra were obtained on a Jasco FT-IR 4100 spectrophotometer (Japan Spectroscopic Corporation, Tokyo, Japan); absorptions are reported in cm^−^^1^. NMR spectra were recorded on a Varian Mercury Plus 400 NMR spectrometer (Varian Inc., Palo Alto, CA, USA) using the residual solvent (CDCl_3_, δ_H_ 7.26 ppm for ^1^H NMR and δ_C_ 77.1 ppm for ^13^C NMR) as the internal standard for ^1^H NMR and CDCl_3_ (δ_C_ 77.1 ppm) for ^13^C NMR. Coupling constants (*J*) are given in Hz. ESIMS and HRESIMS were recorded using a Bruker 7 Tesla solariX FTMS system (Bruker, Bremen, Germany). Column chromatography was performed on silica gel (230–400 mesh, Merck, Darmstadt, Germany). TLC was carried out on precoated Kieselgel 60 F_254_ (0.25 mm, Merck, Darmstadt, Germany); spots were visualized by spraying with 10% H_2_SO_4_ solution followed by heating. Normal phase HPLC (NP-HPLC) was performed using a system comprised of a Hitachi L-7110 pump (Hitachi Ltd., Tokyo, Japan) and a Rheodyne 7725 injection port (Rheodyne LLC, Rohnert Park, CA, USA). Two normal phase columns (Supelco Ascentis^®^ Si Cat #: 581515-U, 25.0 cm × 21.2 mm, 5.0 μm and 581514-U, 25.0 cm × 10.0 mm, 5.0 μm, Sigma-Aldrich. Com. St. Louis, MO, USA) was used for HPLC. 

### 3.2. Animal Material

Specimens of the sponge *Ircinia felix* (Duchassaing and Michelotti, 1864) [16] were collected by hand using SCUBA equipment off the coast of the Southern Taiwan, in September 05, 2012 and stored in a freezer until extraction. A voucher specimen (NMMBA-TWSP-12005) was deposited in the National Museum of Marine Biology and Aquarium, Taiwan.

### 3.3. Extraction and Isolation

Sliced bodies of *Ircinia felix* (wet weight 1210 g) were extracted with ethyl acetate (EtOAc). The EtOAc layer (5.09 g) was separated on silica gel and eluted using a mixture of *n*-hexane and EtOAc (stepwise, 100:1–pure EtOAc) to yield 11 fractions A–K. Fraction F was separated by NP-HPLC using a mixture of *n*-hexane and EtOAc (3:1) as the mobile phase to yield 16 fractions F1–F16. Fraction F4 was purified by NP-HPLC using a mixture of *n*-hexane and acetone (3:1, flow rate: 1.0 mL/min) to afford **1** (1.3 mg, *t*_R_ = 50 min). Fraction G was chromatographed on silica gel and eluted using *n*-hexane/acetone (6:1–2:1) to afford four fractions G1–G4. Fraction G2 was separated by NP-HPLC using a mixture of dichloromethane (DCM) and EtOAc (5:1, flow rate: 2.0 mL/min) to afford **2** (5.8 mg, *t*_R_ = 210 min), **3** (5.3 mg, *t*_R_ = 324 min) and twelve subfractions G2A–G2L. Fraction G2L was further separated by NP-HPLC using a mixture of DCM and acetone (8:1) as the mobile phase to afford **4** (4.4 mg, *t*_R_ = 45 min). Fraction I was separated by NP-HPLC using a mixture of DCM and acetone (4:1) as the mobile phase to afford **5** (3.7 mg, *t*_R_ = 126 min).

Felixin A (**1**): white solid; mp 191–193 °C; ${\left[\alpha \right]}_{D}^{25}$ −84 (*c* 0.4, CHCl_3_); IR (neat) *ν*_max_ 3480, 1731, 1662 cm^−1^; ^1^H (400 MHz, CDCl_3_) and ^13^C (100 MHz, CDCl_3_) NMR data, see Table 1; ESIMS: *m*/*z* 453 [M + Na]^+^; HRESIMS: *m*/*z* 453.29773 (calcd for C_27_H_42_O_4_ + Na, 453.29753).

Felixin B (**2**): white solid; mp 92–94 °C; ${\left[\alpha \right]}_{D}^{25}$ +34 (*c* 0.3, CHCl_3_); IR (neat) *ν*_max_ 3501, 1733, 1679 cm^−1^; ^1^H (400 MHz, CDCl_3_) and ^13^C (100 MHz, CDCl_3_) NMR data, see Table 2; ESIMS: *m*/*z* 467 [M + Na]^+^; HRESIMS: *m*/*z* 467.27707 (calcd for C_27_H_40_O_5_ + Na, 467.27680).

Felixin C (**3**): white solid; mp 194–196 °C; ${\left[\alpha \right]}_{D}^{25}$ +35 (*c* 0.3, CHCl_3_); IR (neat) *ν*_max_ 3480, 1731, 1662 cm^−1^; ^1^H (400 MHz, CDCl_3_) and ^13^C (100 MHz, CDCl_3_) NMR data, see Table 3; ESIMS: *m*/*z* 469 [M + Na]^+^; HRESIMS: *m*/*z* 469.29270 (calcd for C_27_H_42_O_5_ + Na, 469.29245).

Felixin D (**4**): white solid; mp 94–97 °C; ${\left[\alpha \right]}_{D}^{25}$ +22 (*c* 0.2, CHCl_3_); IR (neat) *ν*_max_ 3441, 1738 cm^−1^; ^1^H (400 MHz, CDCl_3_) and ^13^C (100 MHz, CDCl_3_) NMR data, see Table 4; ESIMS: *m*/*z* 525 [M + Na]^+^; HRESIMS: *m*/*z* 525.31849 (calcd for C_30_H_46_O_6_ + Na, 525.31866).

Felixin E (**5**): white solid; mp 151–153 °C; ${\left[\alpha \right]}_{D}^{25}$ −5 (*c* 1.2, CHCl_3_); IR (neat) *ν*_max_ 3421, 1701 cm^−1^; ^1^H (400 MHz, CDCl_3_) and ^13^C (100 MHz, CDCl_3_) NMR data, see Table 5; ESIMS: *m*/*z* 441 [M + Na]^+^; HRESIMS: *m*/*z* 441.29739 (calcd for C_26_H_42_O_4_ + Na, 441.29753).

### 3.4. MTT Antiproliferative Assay

MOLT-4, SUP-T1, DLD-1, LNCaP, T-47D and MCF7 cells were obtained from the American Type Culture Collection (ATCC, Manassas, VA, USA). Cells were maintained in RPMI 1640 medium supplemented with 10% fetal calf serum, 2 mM glutamine and antibiotics (100 units/mL penicillin and 100 μg/mL streptomycin) at 37 °C in a humidified atmosphere of 5% CO_2_. Cells were seeded at 4 × 10^4^ per well in 96-well culture plates before treatment with different concentrations of the tested compounds. The compounds were dissolved in dimethyl sulfoxide (less than 0.02%) and made concentrations of 1.25, 2.5, 5, 10 and 20 μg/μL prior to the experiments. After treatment for 72 h, the cytotoxicity of the tested compounds was determined using a MTT cell proliferation assay (thiazolyl blue tetrazolium bromide, Sigma-M2128). The MTT is reduced by the mitochondrial dehydrogenases of viable cells to a purple formazan product. The MTT-formazan product was dissolved in DMSO. Light absorbance values (OD = OD_570_ − OD_620_) were recorded at wavelengths of 570 and 620 nm using an ELISA reader (Anthos labtec Instrument, Salzburg, Austria) to calculate the concentration that caused 50% inhibition (IC_50_), *i.e.*, the cell concentration at which the light absorbance value of the experiment group was half that of the control group. These results were expressed as a percentage of the control ± SD established from *n* = 4 wells per one experiment from three separate experiments [17,18,19].

## 4. Conclusions

Sponges have been well-recognized as an important source of potential bioactive marine natural products. Our studies on *Ircinia felix* for the extraction of natural substances, have led to the isolation of five new scalaranes, felixins A–E (**1**–**5**) and compounds **1**–**5** are potentially cytotoxic toward DLD-1 tumor cells. These results suggest that continuing investigation of novel secondary metabolites together with the potentially useful bioactivities from this marine organism are worthwhile for future drug development.
